# Targeted DNA methylation from cell-free DNA using hybridization probe capture

**DOI:** 10.1093/nargab/lqac099

**Published:** 2022-12-31

**Authors:** David N Buckley, Gerald Gooden, Kuan Feng, Jacob Enk, Bodour Salhia

**Affiliations:** Department of Translational Genomics, Keck School of Medicine, University of Southern California, Los Angeles, CA, USA; Department of Translational Genomics, Keck School of Medicine, University of Southern California, Los Angeles, CA, USA; Daicel Arbor Biosciences, Ann Arbor, MI, USA; Daicel Arbor Biosciences, Ann Arbor, MI, USA; Department of Translational Genomics, Keck School of Medicine, University of Southern California, Los Angeles, CA, USA; Norris Comprehensive Cancer Center, University of Southern California, Los Angeles, CA, USA

## Abstract

Cell-free (cf)DNA signatures are quickly becoming the target of choice for non-invasive screening, diagnosis, treatment and monitoring of human tumors. DNA methylation changes occur early in tumorigenesis and are widespread, making cfDNA methylation an attractive cancer biomarker. Already a proven technology for targeted genome sequencing, hybridization probe capture is emerging as a method for high-throughput targeted methylation profiling suitable to liquid biopsy samples. However, to date there are no reports describing the performance of this approach in terms of reproducibility, scalability, and accuracy. In the current study we performed hybridization probe capture using the myBaits^®^ Custom Methyl-seq kit on 172 plasma samples and standards to evaluate its performance on cfDNA methylation analysis. The myBaits^®^ assay showed high target recovery (>90%), demonstrated excellent reproducibility between captures (*R*^2^ = 0.92 on average), and was unaffected by increasing the number of targets in a capture. Finally, myBaits^®^ accurately replicated ‘gold standard’ beta values from WGBS (average *R*^2^ = 0.79). The results of this study show that custom targeted methylation sequencing with myBaits^®^ offers a cost-effective, reliable platform to profile DNA methylation at a set of discrete custom regions, with potential applicability to liquid biopsies for cancer monitoring.

## INTRODUCTION

Cell free (cf)DNA signatures are quickly becoming the target of choice for non-invasive screening, diagnosis, treatment, and monitoring of human tumors (1–3). cfDNA arises when DNA fragments from cellular compartments are released into the bloodstream, mainly via apoptosis, cell necrosis, pyroptosis or autophagy. The promise of liquid biopsy based on cfDNA is predicated on the fact that DNA is released by tumor cells into circulation and preserves the molecular characteristics of the tumor from which it was derived (4). In cancer patients, only a small fraction of total cfDNA molecules is derived from the tumor and is referred to as circulating tumor (ct)DNA (5).

CtDNA harbors both the genetic and epigenetic changes of the tumor it is derived from, including single nucleotide variants (SNVs), copy number variants (CNVs) and aberrant DNA methylation. For the purposes of liquid biopsies DNA methylation offers several advantages over other molecular alterations. First, DNA methylation changes occur early in tumorigenesis (6) and are widespread, with thousands to millions of differentially methylated CpG loci between tumor and control tissue (7). The frequency of methylation alterations allows for multiparametric testing, enhancing sensitivity and specificity. Second, aberrant methylation patterns are tissue and cancer-type specific, allowing for early diagnosis from cfDNA (8). Furthermore, methylation patterns can be used to deconvolute tissue of origin in cfDNA (9). Work from Li *et al.* employed a probabilistic approach using cfDNA methylation patterns to identify patients with liver cancer (10). Additionally, recent work has found that methylation patterns can be used to identify tissue specific cell death of cardiomyocytes and pancreatic β-cells indicating this approach has clinical utility beyond cancer detection (11,12).

DNA methylation assays can be generally divided into genome-wide and targeted approaches (13). Genome-wide DNA methylation methods, like whole genome bisulfite sequencing (WGBS), provide single base resolution but are not generally scalable nor cost-effective (14). Targeted methods, however, provide a highly scalable and cost-effective approach to DNA methylation analysis using pre-determined regions of interest. NGS-based targeted approaches have supplemented and, in some cases, supplanted legacy methods such as bisulfite pyrosequencing and methylation specific PCR for measuring methylation at specific genomic targets. An emerging method for targeted methylation profiling is hybridization (hybrid) probe capture, which uses synthetic oligonucleotide probes to retrieve specific sequences from NGS sequencing libraries built from bisulfite- or enzymatically-converted DNA substrate (15,16). Libraries enriched with hybridization capture typically enable high sequencing depth at predetermined genomic loci, which can range from tens-to-millions of CpG sites. While there are examples in literature using commercially-provided hybridization capture kits for the analysis of cfDNA methylation (8,17), these have not comprehensively studied their accuracy compared to gold-standard sequencing, their scalability, or their reproducibility for liquid biopsy applications. myBaits^®^ target capture kits from Daicel Arbor Biosciences provide pools of in-solution biotinylated RNA probes plus reagents for highly efficient, scalable targeted sequencing on any NGS platform. Their methyl-seq hybridization system pairs their unique probe design algorithms with a dedicated target capture protocol to efficiently enrich target genes and markers of interest from methyl-seq NGS libraries. This study is the first to (i) evaluate the performance of myBaits^®^ custom methyl sequencing for assaying cfDNA methylation from blood and (ii) assess the reproducibility, accuracy and scalability of the hybrid probe capture platform from a dedicated technical perspective.

## MATERIALS AND METHODS

### DNA extraction

CfDNA was extracted using the MagMax Cell-Free DNA Isolation Kit (Thermo Fisher) according to the manufacturer's protocol. Genomic (g)DNA was extracted using AllPrep DNA/RNA Mini kit (Qiagen) according to the manufacturer's recommendations. Tissues were homogenized using Bullet Blender homogenizer (Next Advance) for 5 min at full speed with a mixture of 0.9–2.0 mm RNase-free stainless-steel beads. Homogenates were passed through the QIAshredder (Qiagen) to remove any remaining particulate matter. Quantity and purity of the isolated DNA was determined using Tapestation D1000 High Sensitivity tapes.

### Target design of hybrid probe capture cfDNA methylation assays

We used two strategies to design probes; for probeset-1 and probeset-3, we used WGBS read data produced from pools of samples exhibiting a range of methylation states as the reference sequence for probe design. Raw 151 nt reads that aligned to the target genome regions were extracted from source BAM files, and then 80 nt probe sequences were tiled across these reads at roughly 3× tiling density (i.e. three 80 nt probes per read). This set of initial candidates were then aligned to each other and collapsed to reduce their overall number. For probeset-1 probe candidates (starting = 458K candidate probes), any probe that overlapped with another by 60 nt or more and that exhibited 95% or higher sequence identity in that overlap was removed (final representative probes = 32K). For probeset-3 probes (starting = 222K candidates), probes aligning to other probes by 78 nt or more and exhibiting 97% sequence identity within their overlap were removed (final representative probes = 75K).

Sequence diversity in the WGBS dataset for the probeset-2 targets was particularly high. Applying the WGBS-based probe design strategy for these targets resulted in a significantly larger probe design than for probset-1 and probeset-2 targets (350–550K total probes even after specificity filtration and sequence compression). Rather than down-sample the WGBS data to a smaller set of representatives, we opted to use Daicel Arbor's proprietary in silico design strategy for these targets. Briefly, the procedure begins by simulating some but not all potential methylation states for a given sequence space, selects probes for these simulated sequences, and then filters the probes for locus specificity. This resulted in a probe set with a total number of probes more comparable to the other sets—roughly 58K candidate probes. Once all candidates were selected, they were filtered for specificity using Arbor's standard filtration regimen. Candidates were kept if they passed Arbor's ‘relaxed’ filtration level when screened against fully CpH > TpH converted versions of both genome strands of hg19. This resulted in final probe sequence counts of 19 155 for probeset-1, 44 928 for probeset-2 and 68 966 for probeset-3.

### Hybrid probe capture: library preparation, target enrichment and bisulfite sequencing

In total, we performed hybrid probe capture on cfDNA from 172 cfDNA samples (see Supplementary Table S1 for details). Of these, 28 samples were re-run to assess technical and biological reproducibility. cfDNA samples were purchased from commercial sources (Discovery Life Sciences), the Molecular Pathology Core at the University of Southern California, or Memorial Sloan Kettering Cancer Center. In addition, we generated a 6-point standard curve by mixing 0% and 100% enzymatically generated methylation technical controls using gDNA (Zymo Research) fragmented down to ∼160 bp via sonication to simulate cfDNA. The resultant standards consisted of 0%, 7%, 15%, 25%, 50% and 100% methylated DNA samples used to assess whether the probe sets could accurately reproduce the known starting methylation levels of each standard. Standards were run in duplicate for probeset 2 and 3 and quadruplicate for probeset 1 to assess biological and technical reproducibility. We used 10 ng of extracted cfDNA for bisulfite treatment using the EZ DNA Methylation-Gold Kit (Zymo Research), followed by library preparation with the Accel-NGS Methyl-Seq DNA Library Kit (Swift Biosciences) and 11 cycles of indexing amplification using unique dual 8bp indexing primers. Based on the Bioanalyzer trace, between 23 and 90% of the total sequencing library molecules fell between 200 and 650 bp, in which the true cfDNA fraction is expected to fall (18). Eight or more libraries were pooled for each enrichment reaction, with a total library mass of up to 1.6 ug insert-containing templates. Target enrichment was carried out on 200 ng of library following the myBaits version 5 High Sensitivity protocol (Daicel Arbor Biosciences) using either a single probe set (e.g. probeset-1, probeset-2 or probeset-3) or a pool of all three probe sets (probeset-all). Briefly, bisulfite-converted DNA libraries were incubated with biotinylated probes and adapter blockers in hybridization buffer overnight at 63°C. Probe-bound libraries were pulled down with streptavidin beads followed by four 63°C washes and amplified with 14 PCR cycles and purified with solid phase reversible immobilization (SPRI). These products were then enriched again to achieve high target capture efficiency. The final enriched libraries were quantified with KAPA Library Quantification Kit (Roche) and sequenced on a NovaSeq using 2 × 150 cycle and 2 × 100 cycle runs. Several captures were also sequenced using PE75, PE150 and PE300 protocols with a MiSeq using v3 chemistry. In all cases, libraries were demultiplexed using both index 1 and index 2 and read ends were trimmed according to Swift's recommended protocol for libraries prepared with the Accel-NGS kit. We performed 27 separate captures in total, referred to here as SC01 to SC27 (Table 1).

**Table 1.** Capture summary: table summarizes all captures (SC01–SC27); which probeset each capture used, and the sequencer/chemistry used

Capture	Probeset	Platform
SC01	PROBESET-1	NovaSeq_PE150
SC02	PROBESET-1	NovaSeq_PE150
SC03	PROBESET-1	MiSeq_PE75 - NovaSeq_PE150
SC04	PROBESET-1	MiSeq_PE75 - NovaSeq_PE150
SC05	PROBESET-1	NovaSeq_PE150
SC06	PROBESET-1	NovaSeq_PE150
SC07	PROBESET-ALL	MiSeq_PE75 - MiSeq_PE300
SC08	PROBESET-2	MiSeq_PE75 - NovaSeq_PE150
SC09	PROBESET-1	NovaSeq_PE150 - MiSeq_PE75
SC10	PROBESET-2	NovaSeq_PE150
SC11	PROBESET-ALL	NovaSeq_PE150
SC12	PROBESET-ALL	NovaSeq_PE150
SC13	PROBESET-2	NovaSeq_PE150
SC14	PROBESET-1	MiSeq_PE300
SC15	PROBESET-3	MiSeq_PE150
SC16	PROBESET-3	NovaSeq_PE100
SC17	PROBESET-3	NovaSeq_PE100
SC18	PROBESET-3	NovaSeq_PE100
SC19	PROBESET-3	NovaSeq_PE100
SC20	PROBESET-3	NovaSeq_PE100
SC21	PROBESET-3	NovaSeq_PE100
SC22	PROBESET-3	NovaSeq_PE100
SC23	PROBESET-3	NovaSeq_PE100
SC24	PROBESET-3	NovaSeq_PE100
SC25	PROBESET-3	NovaSeq_PE100
SC26	PROBESET-3	NovaSeq_PE100
SC27	PROBESET-3	MiSeq_PE150

In order to evaluate reproducibility, we compared sample-matched sequencing runs from the same capture (termed *intra-*capture) and sample-matched sequencing runs that were captured two or more times (termed *inter*-capture). In addition to entirely new libraries, we also captured 23 libraries that had previously been sequenced using WGBS (Supplementary Table S1) in order to measure the degree of correlation of DNA methylation using WGBS and hybrid probe capture.

### Next generation sequencing data analysis

Paired end FASTQ files were generated on MiSeq and NovaSeq sequencers (Illumina). After demultiplexing, FASTQ quality was assessed using FastQC. Based on results from FastQC FASTQs from SC14 were hard trimmed at the 3′ end from 300 to 100 bp. After QC, FASTQ adapter trimming was performed using TrimGalore. For hybrid probe capture samples, read 2 FASTQs were trimmed 10bp from the 5′ end to remove the low complexity oligonucleotide introduced by Swift Biosciences’ adaptase. After trimming, paired end reads were mapped to hg19 using Brabham Bioinformatics’ Bismark BS-seq alignment software (19). After alignment duplicate reads were removed using Samblaster (20). Methylation per CpG was evaluated using Bismark's methylation extractor tool. QC reports were combined using MultiQC (21). All downstream analysis was performed in R using the bsseq package (22).

## RESULTS

### Sequencing metrics

**Figure 1.**
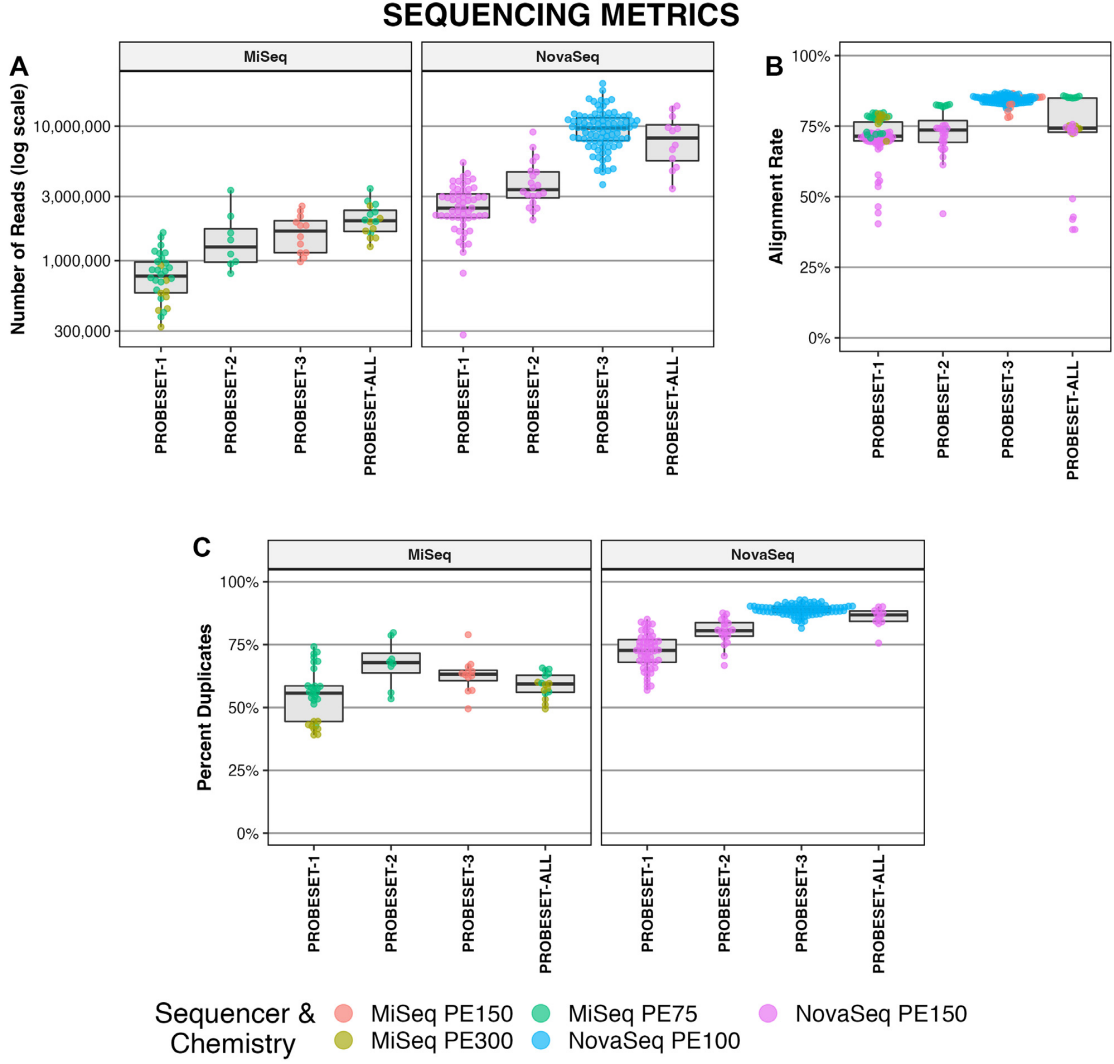
Sequencing metrics from hybrid probe capture cfDNA samples. (**A**) Number of paired reads output per sample. Samples are subdivided by probeset (1–3 & all) and sequencing platform (MiSeq & NovaSeq). Note: y-axis is scaled logarithmically. (**B**) Bismark alignment rate per sample and probeset. (**C**) Percent of reads evaluated as PCR or optical duplicates. All plots are colored by sequencer and chemistry as noted in legend.

After demultiplexing we used FastQC to quantify the number of raw reads per sample, mean quality score along the length of each read, and adapter content of each read pair. Quality scores along the length of each read were >30 (Phred-score) in all the sequencing runs except the MiSeq paired-end 300 (600 cycle) and NovaSeq paired-end 150 (300 cycle) sequencing runs (Supplementary Figure S1). This is due to the fact that cfDNA fragments are generally ∼160 bp long (23) and sequencing fragments to 300 bp on the MiSeq results in sequencing the Illumina adapter itself at the 3′ end of each read (Supplementary Figure S2). This was also observed toward the end of shorter fragments on our NovaSeq PE150 run. On average, samples had 2.8 million paired reads with a range of 280 000 to 14M paired reads (Figure 1A). Overall, 73% of reads mapped uniquely (Figure 1B) which is a typical alignment rate for bisulfite treated DNA aligned with Bismark software. On average, 76% of reads were PCR duplicates and were removed (Figure 1C).

### Hybrid probe capture shows high target representation and coverage

**Figure 2.**
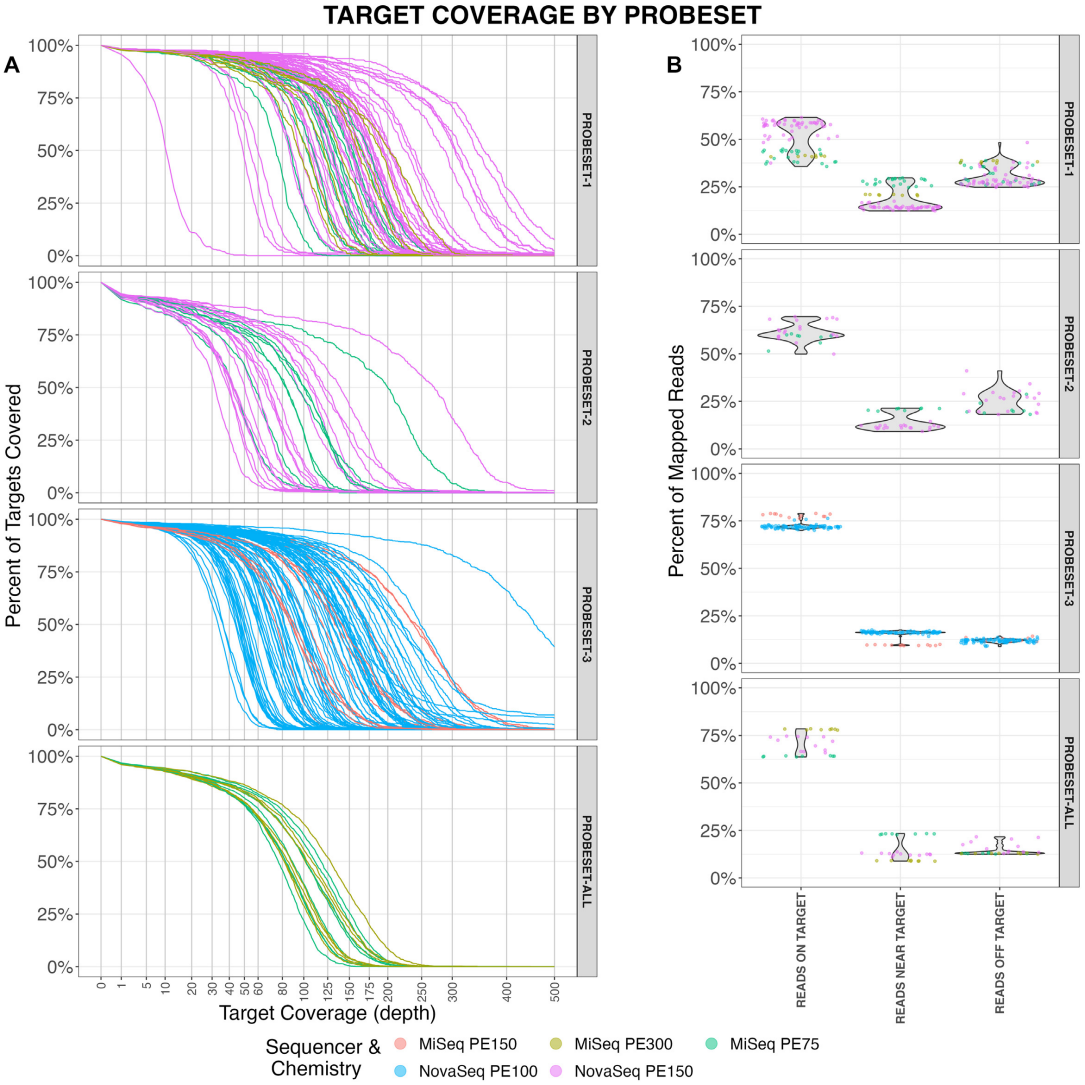
Hybrid probe capture shows high target coverage, percent of targets covered, and on target reads. (**A**) Coverage plots (by sample, color-coded by sequencer & chemistry) of percent of targets in a given probeset covered at a given depth (*x*-axis). Plots are subdivided by probeset. (**B**) Percent of reads mapped to target regions, near target regions (±300 bp) and off target (>300 bp from a target region). A buffer of 300 bp was used for ‘reads near target’ as the probe capture design was slightly larger than the target regions.

We were primarily interested in the percent of targets covered and the average sequencing depth in each target region. We define a ‘target’ as the original DMR call we found during feature selection; the probe capture design was padded ∼100 bp upstream and downstream of the target to ensure high coverage across the entire target region (Supplementary Figure S3). Overall, we found that myBaits had high target recovery and sequencing depth across the board. All probesets had a vast majority of targets represented with very few targets dropping out entirely (Figure 2A); in probesets-1–3 and probeset-all only 1.9%, 6.6%, 1.5% and 3.6% of targets had <1× average coverage, respectively. The proportion of targets covered at 10× or greater was 96%, 89%, 96% and 93% for probesets-1–3 and probeset-all, respectively. The median target coverage of each of these probesets was 142.2×, 64.9×, 95.1× and 93.6×. We additionally wanted to quantify the fraction of reads mapped to target regions to evaluate the efficiency of the capture. The design of the probeset was slightly larger than the actual target regions, thus we included ‘reads near target’ – reads mapped within 300bp of a target region – to account for this padding. On average, 64% of reads were mapped to a target region, however, this did vary by probeset (Figure 2B). Probesets-1, 2, 3 and probeset-all had 51%, 61%, 73% and 71% of reads on target, respectively.

### Target coverage shows consistent performance

**Figure 3.**
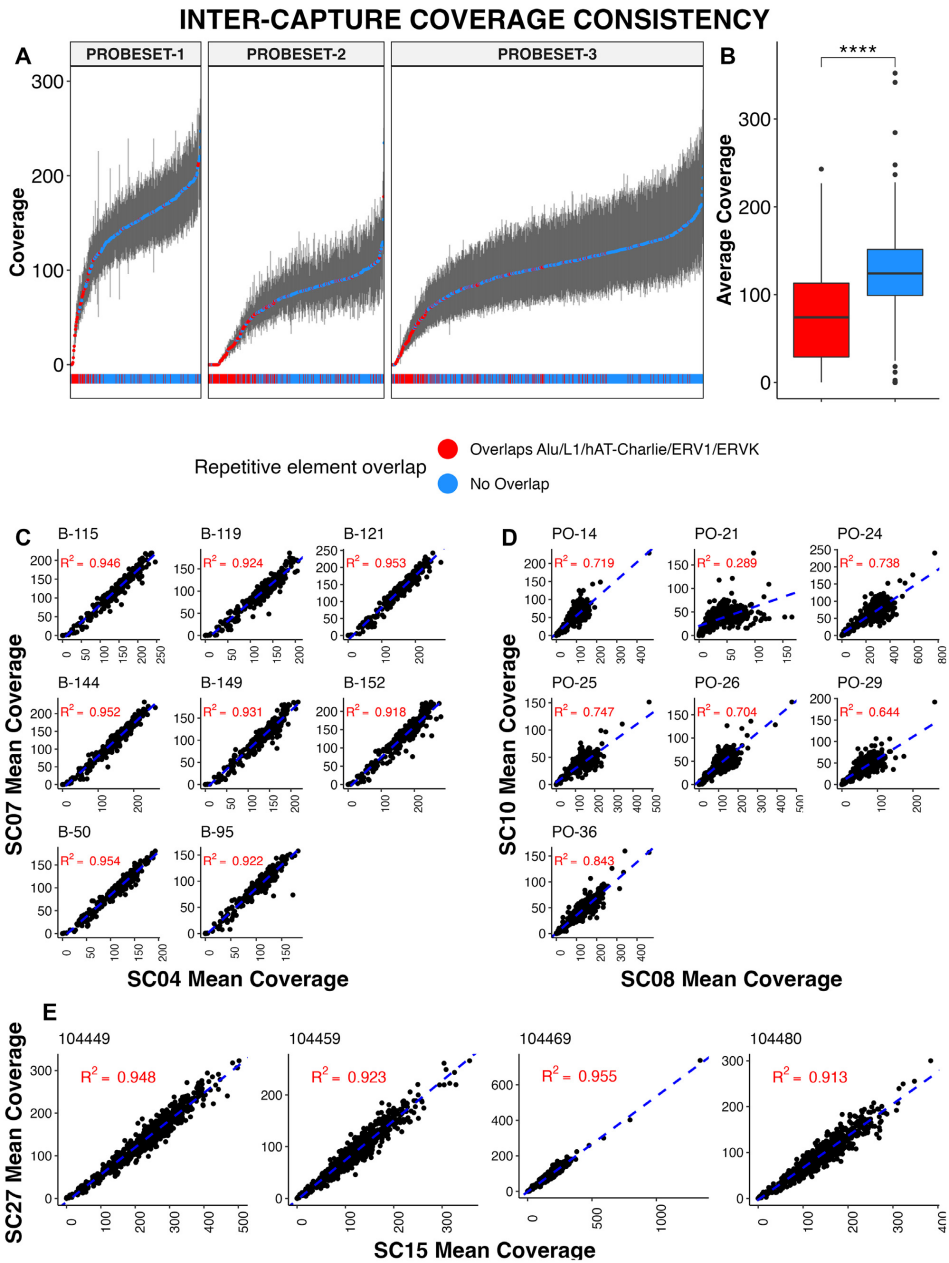
Hybrid probe capture demonstrates high coverage consistency in target regions. (**A**) Plot of median coverage (in blue, with target regions overlapping repetitive elements (Alu/L1/Hat-Charlie/ERV1/ERVK) highlighted in red, and inter-quartile ranges (IQR) indicated by grey vertical lines), across all runs and all samples per target region. The relatively narrow IQRs indicate coverage consistency across multiple samples and runs. (**B**) Boxplot showing target coverage in repetitive elements compared to outside of repetitive elements. (**C–E**) Scatterplot comparisons of mean target coverage between two captures (inter-capture), for matched patient samples. Plots are grouped by probeset: C = probeset-1, D = probeset-2 and E = probeset-3. Line of best fit is indicated by the blue dashed line.

In order to assess whether DMRs are consistently covered by the myBaits hybrid capture method, we first determined the median coverage and inter-quartile range (IQR) for each target across all samples and all sequencing runs per target region (Figure 3A). We found that regions with low coverage commonly overlapped repetitive elements: Alu, L1, ERV1, ERVK and hAT-Charlie were all found to be significantly associated with a decrease in target coverage (ANOVA *P*-value < 0.001 for all). The average coverage of targets containing at least one of these elements was 74× compared to 126× in targets that did not contain a repetitive element (Figure 3B) (Student's *t*-test *P*-value < 0.00001). These repetitive elements are represented multiple times in the human genome and are a relatively common confounding factor in multiple genomic applications. While repetitive elements help to explain the relative performance in a majority of poorly performing target regions, there are exceptions—targets that overlap repetitive elements that perform well and those that do not perform poorly. Thus, filtering out targets overlapping repetitive elements prior to capture design can be used to mitigate the proportion of targets with low coverage but will likely not eliminate all of them.

We also wanted to evaluate inter-capture target coverage—results from two separate captures of the same samples. To evaluate inter-capture target coverage from the same library we ran 8 samples from SC04 (probeset-1) and SC07 (probeset-all). Both captures were performed on the same library, and both were sequenced on the MiSeq platform (PE 75). We then compared per-target coverage between the two captures (Figure 3C). Additionally, despite the addition of 1517 targets in SC07 (probeset-all) the overall per-region coverage was only minimally affected; the average target coverage in SC07 was 109× compared to 132× in SC04; a diminution of only 16% despite an increase in total target size of 91%. Next, we evaluated target coverage between two separate libraries by comparing SC08 versus SC10 (both probeset-2) and SC15 versus SC27 (both probeset-3). As with SC04 versus SC07, the same samples were captured twice. Target coverage in SC08 and SC10 was well correlated, however the correlation was not as strong as SC04 versus SC07 (Figure 3D). *R*^2^ values ranged from 0.29 to 0.84, with significantly lower coverage in SC10 (45×) compared to SC08 (114×). Target coverage in SC27 compared to SC15 was highly correlated (all *R*^2^ values > 0.9) (Figure 3E). Average target coverage in SC15 was 163× compared to 101× in SC27. The decrease in overall coverage in SC27 is likely due to the fact that there were 12 additional samples (not presented here) run alongside SC27, competing for space on the flow cell.

### Accuracy and linearity in beta value measurements using fragmented genomic DNA standards

**Figure 4.**
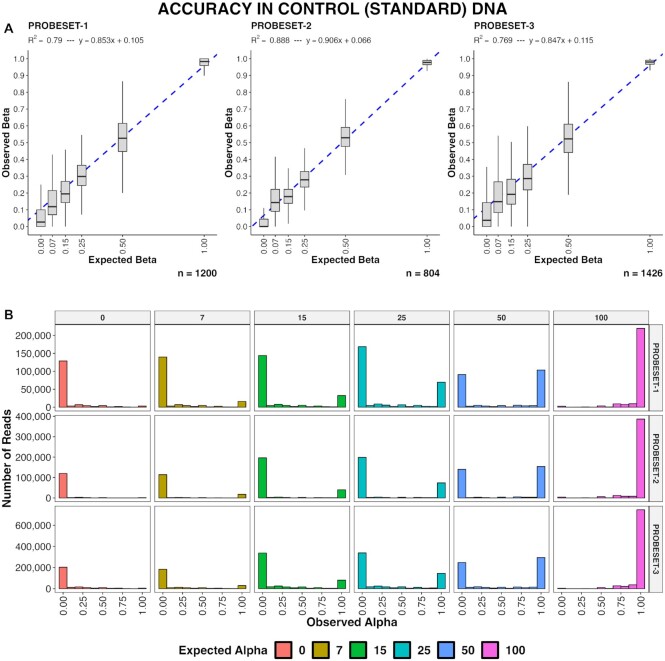
Standard curves and technical control DNA. (**A**) Linearity of observed beta values against expected, in synthetic admixtures with increasing percent methylation (0%, 7%, 15%, 25%, 50% and 100%), shown by probeset, with linear regression models shown in blue. The number (n=) of individual standard curves represented by each plot are shown (bottom right). (**B**) Histogram is plotting alpha values of each methylation control subdivided by probeset (rows) and expected beta values of each control (columns). The x-axis is the observed alpha value and the y-axis is the number of reads for a given alpha value. Alpha values evaluate the average methylation across the read.

We next wanted to determine whether there is methylation quantitation bias towards methylated or unmethylated DNA with the probes we designed. To do this, we generated a 6-point standard curve by mixing 0% and 100% enzymatically generated technical methylation controls. In total, 3430 standard curves were generated across multiple captures and all three probesets. For this, curves were combined per probeset to evaluate overall behavior (Figure 4A). All three probesets produced methylation levels very similar to the expected levels—*R*^2^ values for each of the three probesets were 0.79, 0.89 and 0.77 respectively. All three probesets consistently evaluated the 100% methylated technical control at or very near 100%; mean observed beta value of the 100% technical control in each of the three probesets was 95.7%, 96.7% and 97.1%, respectively. Mean observed beta value in the 0% technical control was 9.4%, 3.8% and 11.4%. The alpha value of each read – that is the average methylation across the read—were also tabulated (Figure 4b). The proportion of methylated to unmethylated DNA was as expected with no evidence for a bias towards methylated or unmethylated species.

### Hybrid probe capture correlates with WGBS

**Figure 5.**
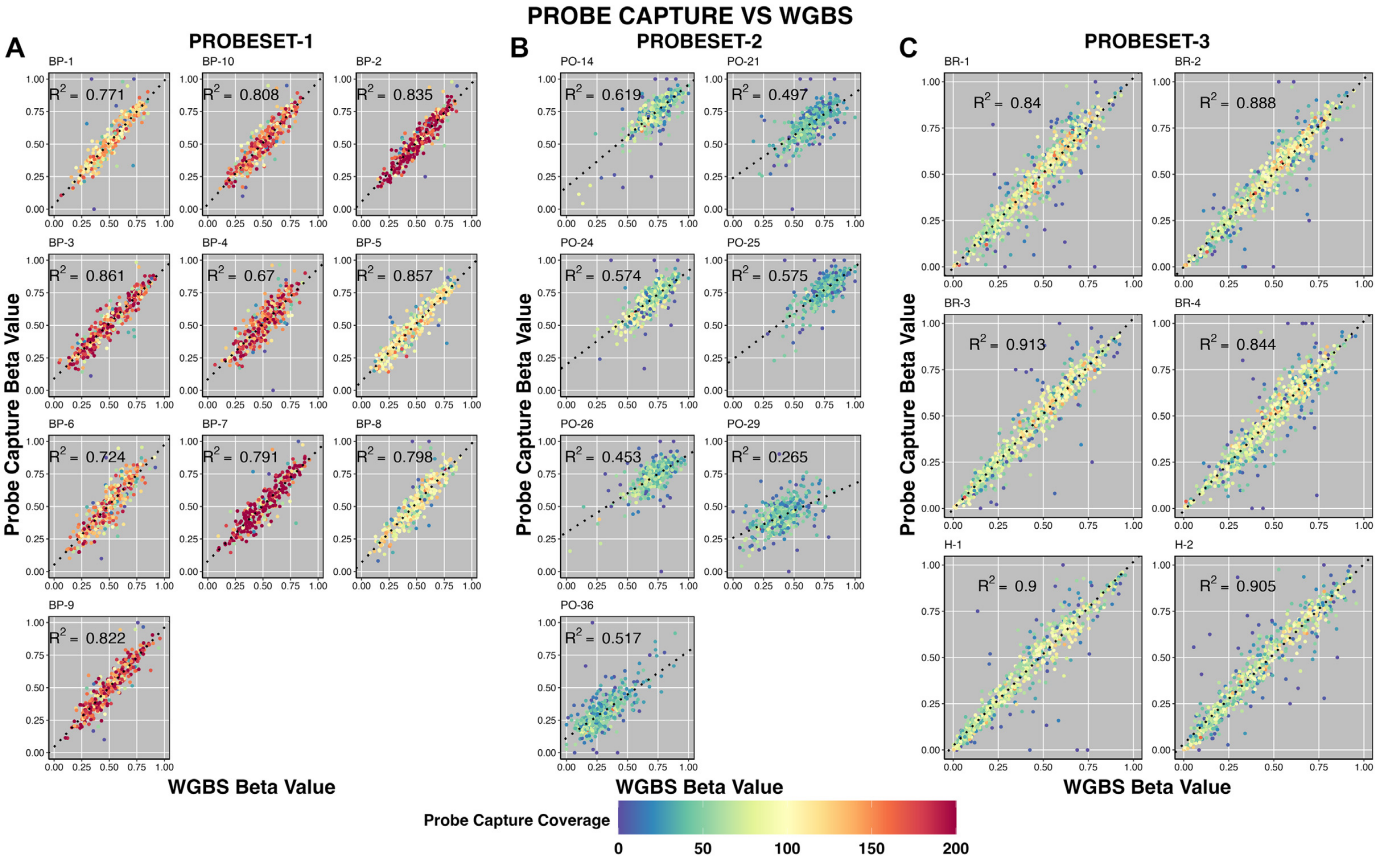
Hybrid probe capture correlates with beta values determined by WGBS. (**A–C**) Scatter plots comparing hybrid probe capture beta values (*y*-axis) with WGBS beta values (*x*-axis). Figure is grouped by sample and probeset. Points are colored by target coverage in hybrid probe capture. Line of best fit is indicated by the block dots.

In order to assess the correlation of hybrid capture beta values with WGBS, we tested a total of 23 libraries that were previously sequenced using WGBS: 10 with probeset-1, 7 with probeset-2 and 6 with probeset-3. For probeset-1, beta values from hybrid probe capture correlated strongly with beta values from WGBS across the 300 target regions. The average *R*^2^ value for all 10 samples was 0.79 (min = 0.67, max = 0.86) (Figure 5A). The slopes of the linear models were 0.90 on average ranging from 0.85 to 0.95. As previously observed, beta values in seven samples captured using probeset-2 clustered in a condensed group because they have a skewed distribution (Figure S4) owing to increased hypermethylation in these samples. The average *R*^2^ was 0.50 (min = 0.27, max = 0.62), and the average slope of the linear models was 0.65 (min = 0.41, max = 0.78) (Figure 5B). Beta values in six samples captured using probeset-3 correlated highly with data obtained by WGBS. The average *R*^2^ was 0.88 (min = 0.84, max = 0.91); the average slope was 1.01 (min = 0.97, max = 1.03) (Figure 5C). Combining all samples from all probesets yielded an *R*^2^ of 0.86 with a slope of 0.95. Taken together, these results show hybrid probe capture accurately replicates beta values of WGBS.

### Hybrid probe capture is consistent between intra and inter-capture comparisons

**Figure 6.**
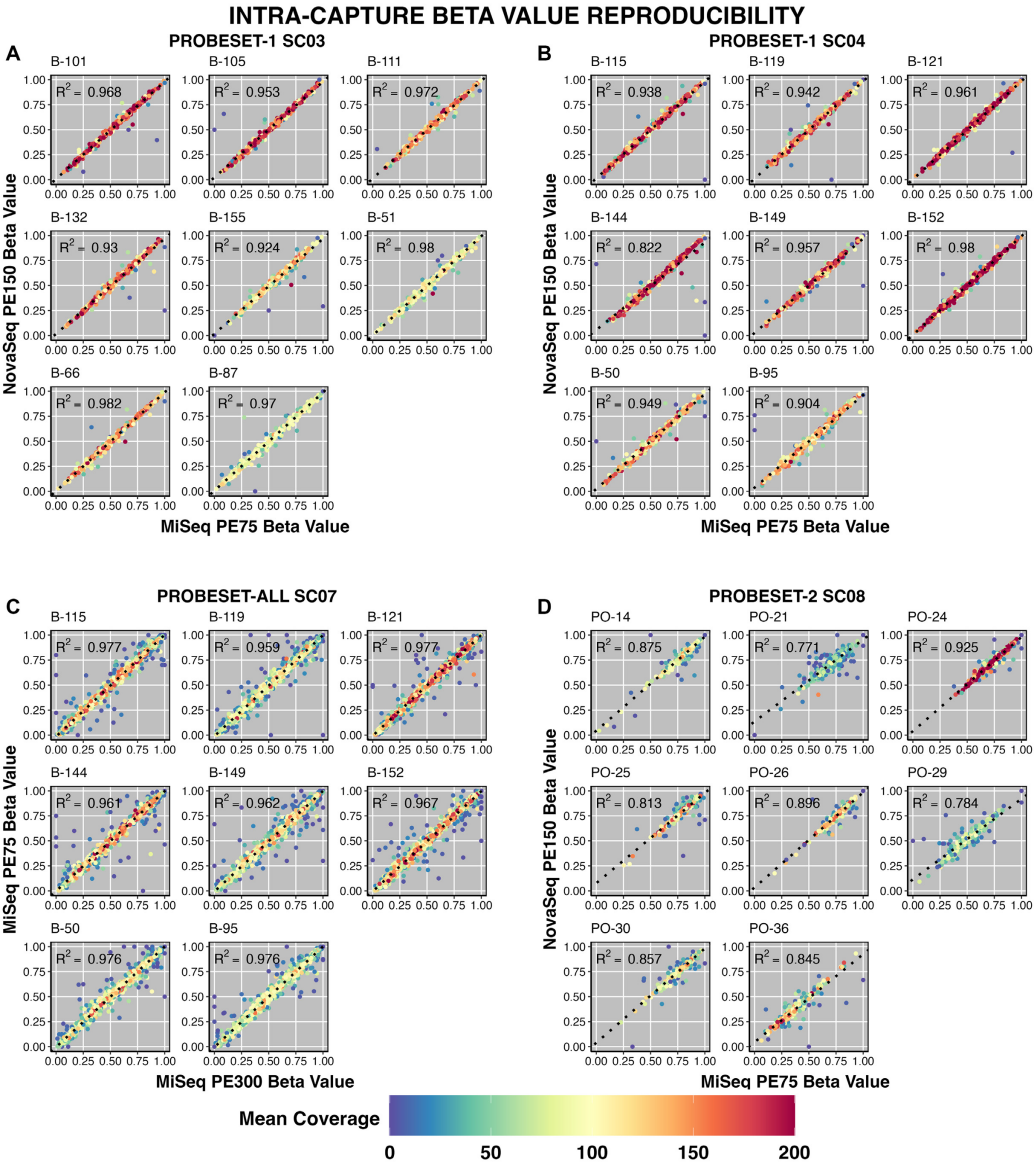
Hybrid probe capture shows high beta value consistency intra-capture & between sequencing platforms. (**A–D**) Scatter plots comparing samples from the same capture run on two different sequencing platforms, as indicated to the left and bottom of each sub-figure, with samples grouped by probeset. Points are colored by average target coverage between the two sequencing runs; those that deviate from linearity tend to have low coverage (blue). Line of best fit is indicated by the block dots.

To assess the reproducibility of the hybrid capture method for cfDNA methylation analysis, we performed a series of hybrid captures between different library preparations and captures (inter-capture) and between captures sequenced more than once (intra-capture). For this, we sequenced 4 captures (SC03, SC04, SC07 and SC08), twice to compare intra-capture beta value consistency, as well as consistency using different sequencers/chemistry. Each capture was hybridized with different probesets as shown in Supplementary Figure S5 and consisted of 8 samples each. SC03, SC08 and SC04 captures were run on both a MiSeq using PE 75 and a NovaSeq with PE 150 chemistry. SC07 capture was sequenced on MiSeq PE300 and a MiSeq PE75. Beta values per target region were compared between each sequencing run (Figure 6). Correlation of beta values between samples in intra-capture runs was very high (Figure 6A): SC03 (range 0.92–0.98, average *R*^2^ = 0.96); SC04 *R*^2^ ranged from 0.82 to 0.98 with an average *R*^2^ of 0.93 (Figure 6B). SC07 (probeset-all) was run twice on a MiSeq with PE 75 and PE 300 chemistry. Correlation remained high with *R*^2^ values ranging from 0.96 to 0.98 (Figure 6C). Finally, we compared two runs of SC08 (probeset-2). *R*^2^ values ranged from 0.77 to 0.92 (Figure 6D)—moderately lower than the previous comparisons. The lower correlation of probeset-2 is due to a skewed distribution of beta values (Supplementary Figure S4), concentrating data points at one locus and decreasing the ability of a linear model to accurately evaluate correlation. However, we see a similar skewed beta value distribution in in WGBS (detailed above) data from the same samples and is therefore likely due to limited biological beta value variation, not a bias of the probe capture assay.

**Figure 7.**
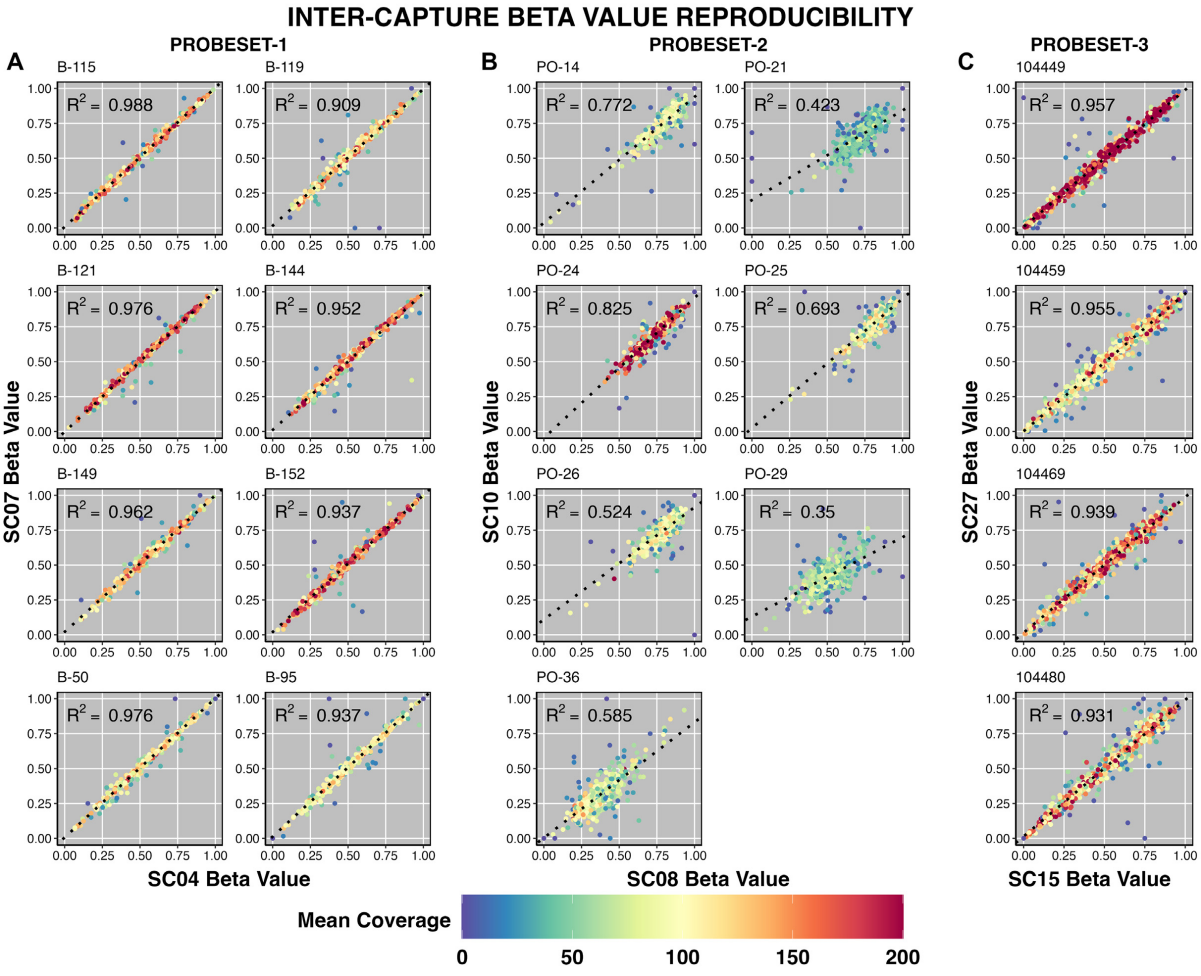
Hybrid probe capture shows high beta value consistency inter-capture. (**A–C**) Scatter plots comparing samples captured multiple times. The capture for each axis is annotated on the left and bottom of each sub-figure, with samples grouped by probeset. Points are colored by average target coverage between the two sequencing runs; those that deviate from linearity tend to have low coverage (blue). Line of best fit is indicated by the block dots.

Having established that there is very little deviation in beta value intra-capture, we wanted to establish how beta value correlates between captures of identical libraries, as well as of separate library preparations of the same patient sample. As with the inter-capture target coverage comparisons above, we compared SC04 to SC07 (same library, probeset-1 and probeset-all respectively), SC08 to SC10 (same library, probeset-2 for both) and SC15 to SC27 (different libraries, probeset-3 for both). Inter-capture beta value correlation for 8 common samples in SC04 and SC07 was comparable to that observed intra-capture, with *R*^2^ values ranging from 0.91 to 0.99 with an average *R*^2^ of 0.95 (Figure 7A). As with intra-capture beta value correlation, regions that deviated from perfect correlation had comparatively low coverage. The seven common samples in SC08 and SC10 had lower correlations; *R*^2^ values ranged from 0.35 to 0.83 with an average of 0.60 (Figure 7B). Again, the skewed beta values in samples captured using probeset-2 affected inter-capture correlation. SC15 and SC27 had only four common samples, however, the correlations of these samples was also very high with an average *R*^2^ value of 0.95 – ranging from 0.93 to 0.96 (Figure 7C). Notably, SC15 and SC27 used separate bisulfite conversions and library preparations for each capture, and demonstrated comparable *R*^2^ values to SC04 and SC07 which were captured using the same library preparation.

### Scalability of hybrid probe capture

**Figure 8.**
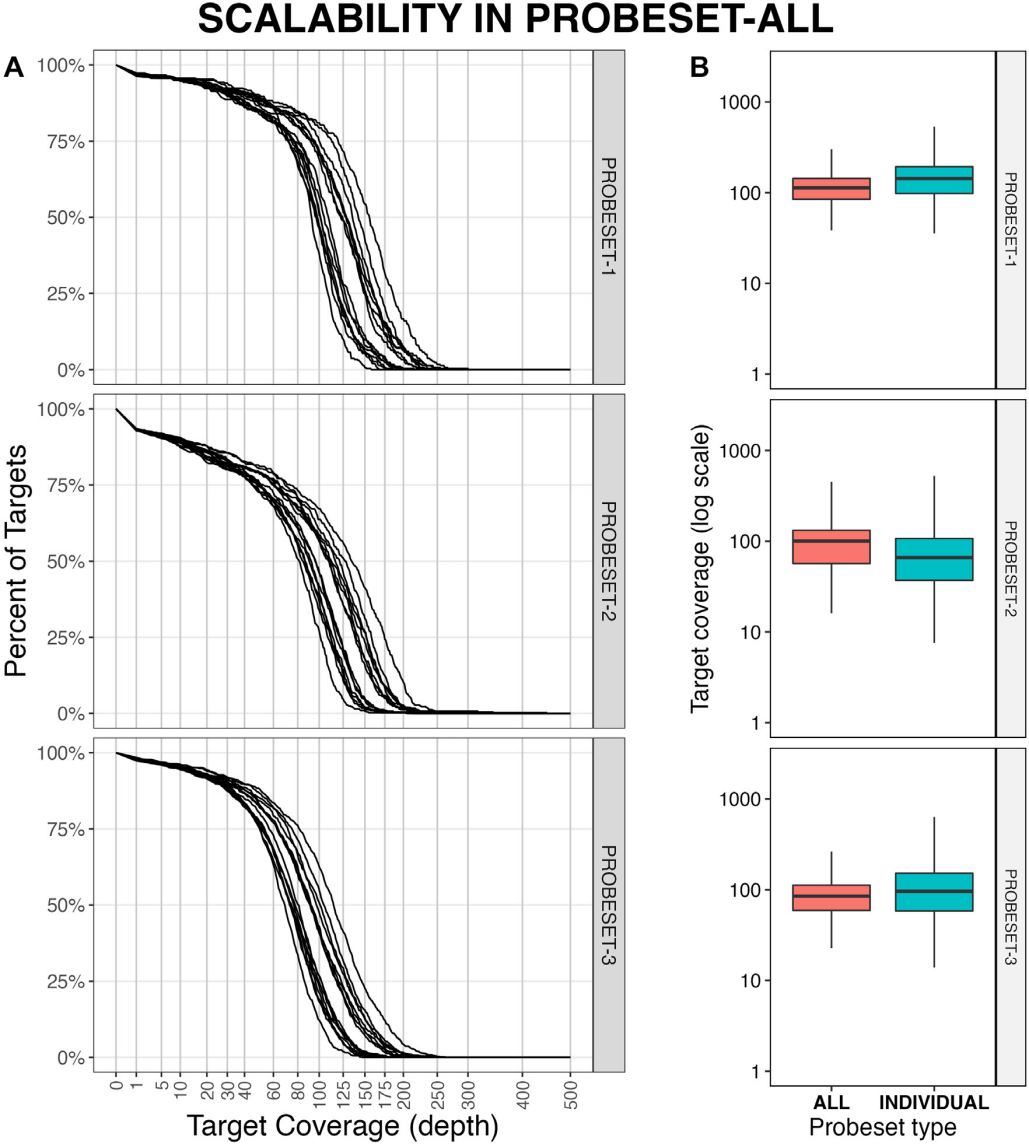
Combining probesets does not have a dramatic effect on target coverage. (**A**) Coverage plots of the target coverage, by probeset, showing close similarity between probesets. (**B**) Comparison of average target coverage in samples run on probeset-all (left) and samples run on individual probeset (right), by probeset, again indicating consistent coverage.

In order to examine how expanding the total target space in a single hybridization capture reaction affects the efficiency of retrieving all targets, we also performed captures using a pool of all three probe sets (probeset-all). One capture, SC07, was performed on 8 patient samples and sequenced twice (MiSeq PE300 & PE75). Overall, target coverage breadth and depth (after read depth normalization) using probeset-all was comparable to coverage obtained with captures using the individual probesets (Figure 8A). We did detect some differences: average target coverage between probeset-all and each respective individual probeset; average target coverage for probeset-1 within probeset-all was 110× compared to 151× when running probeset-1 alone, 93× compared to 79× in probeset-2 and 85× compared to 113× in probeset-3 (Figure 8B). Overall, target coverage in probeset-all was lower compared to individual probesets, however, the coverage difference was not significant enough to affect beta value consistency (Figure 7A). This comparable coverage breadth and depth was achieved with ∼2M reads per sample in probeset-all, compared to 1.9M, 3.3M and 8.8M reads in probeset-1, 2 and 3, respectively.

### Hybrid probe capture performs consistently across multiple genomic contexts

**Figure 9.**
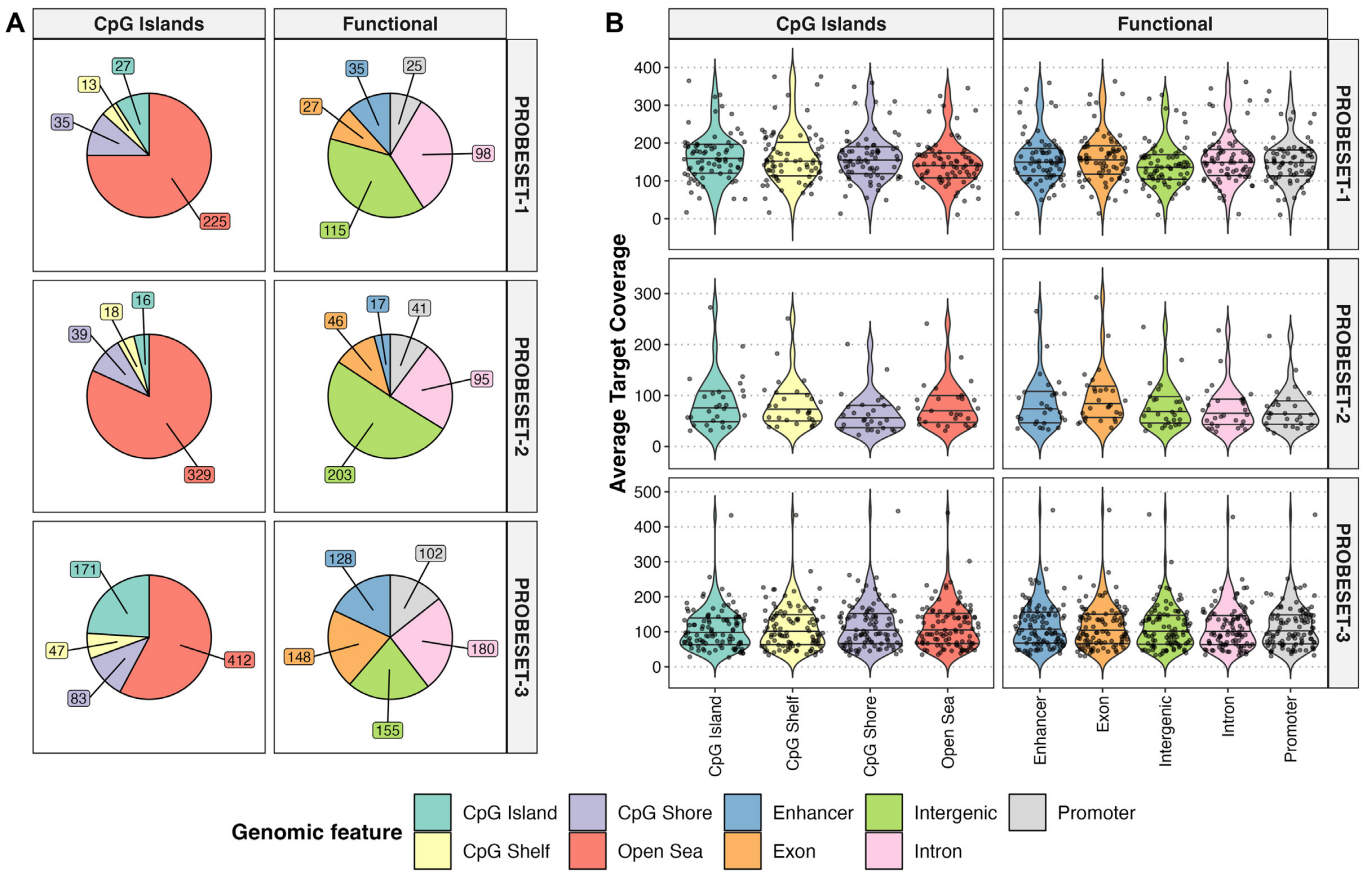
Comparison of target coverage across multiple genomic contexts. (**A**) Pie chart distribution of the genomic location of all DMR targets per probeset, according to proximity to CpG islands and functional contexts. The number in each pie slice represents the number of DMRs represented in that feature. (**B**) Violin plots showing the average target coverage per sample in each genomic context by probeset. The black lines in each violin plot represent the interquartile range.

Finally, we wanted to examine if genomic context introduced any bias in performance of hybrid probe capture. The DMRs used to define our target regions were derived from multiple cancers and were comprised of features across multiple genomic contexts. The genomic context of the DMRs in each probeset included CpG islands/shores/shelves/open sea, as previously defined (24) and functional regions (promoters, exons, introns, and enhancers and intergenic) (Figure 9A). To assess the impact of a target's genomic context by probeset, we used target coverage as the metric for performance. Mean coverage was calculated for each target and sample and averaged across the different genomic context per probeset (Figure 9B). We found no significant difference in coverage between CpG islands, CpG shores, CpG shelves and open sea (ANOVA *P*-value > 0.30 for all probesets). We also did not find any significant difference in coverage for functional features (ANOVA *P*-value > 0.35 for all probesets). Taken together, the data show that hybrid probe capture for cfDNA methylation analysis is unaffected by genomic context when assessing different DMRs in different probesets.

## DISCUSSION

The use of cfDNA methylation in liquid biopsies has shown great potential for molecular testing (13,25–29). Previous work by Lasseter *et al.* (30) found that cfDNA methylation (using MeDIP-seq) was far more sensitive and specific than cfDNA SNV markers in detecting renal cell carcinoma. There are numerous microarray and NGS-based assays already available to detect and measure DNA methylation on a genome-wide basis (31). However, a targeted assay is only useful if it can accurately retrieve sequence information supplied by gold standard methods (here WGBS) while significantly reducing the costs of obtaining that information. Here we evaluated the accuracy, reproducibility, and scalability of a probe hybridization capture platform for methylation sequencing of cfDNA (myBaits® Methyl-Seq). Hybridization capture technology is particularly well suited to cfDNA applications because not only is it tolerant of moderate levels of sequence mismatch, but it is also capable of simultaneously retrieving multiple loci from rare, highly fragmented DNA templates imbedded in complex backgrounds, which is typical of cfDNA. These are also conditions that challenge legacy targeted bisulfite sequencing platforms. We found that the myBaits system accurately reproduces the methylation signals in cfDNA obtained via WGBS, that it does so consistently between experiments and for the same samples prepared in different ways, and finally that it is robust to variation in target sizes.

We found that custom myBaits probe sets were both specific and sensitive ‘right out of the box’. First, the percent of targets recovered was very high, with >90% of targets represented at 10× or greater coverage; very few regions dropped out entirely, which is essential when moving from feature discovery to validation. Second, we did not find evidence of methylation bias in captured sequences from technical control DNA samples, or when comparing hybrid probe capture to WGBS. Although methylation bias is a problem that can be corrected *in vitro* and *in silico* (32), it is preferable to not have to apply any correction after sequencing. This implies that the probe design strategies we employed were effective at minimizing allelic capture bias. Further, since hybrid capture is performed on NGS libraries built from randomly fragmented genomic DNA, PCR duplicate reads can be removed that might otherwise obscure true methylation levels in a sample. Third, hybrid probe capture allows profiling of large collections of genomic targets simultaneously in a single reaction which is highly cost and time efficient. By contrast, targeted amplicon sequencing requires tiling primer pairs across genome regions of interest, thus requiring multiple individual reactions or carefully designed multiplex reactions to reconstruct just a single region. Not only is this traditional targeted sequencing approach more laborious, but most deployments of this technology also consume more raw cfDNA material per assay. This is particularly significant when analyzing cfDNA as the total yield from peripheral blood can be extremely small and often only enough to assay once before the sample is exhausted.

While we identified and validated numerous benefits to the hybrid capture platform, it does have some shortcomings. First, the assay requires not just the generation of sequencing libraries, but also multiple steps where samples must be pooled, then enriched in two successive rounds, each of which involves multiple cycles of PCR and thus introduces coverage biases. We did not evaluate the target coverage potential after just one round of enrichment, but it is possible that it would be sufficient to still retrieve the target in a reasonable amount of sequencing. Second, the percentage of raw reads on-target with these custom designs can be relatively low compared to more developed hybridization capture assays, with probeset-1 being a good example of this (mean 51% on-target). Consequently, much of the flow cell output for such low-specificity assays are non-informative. Compounding this is the fact that repetitive elements decrease the pulldown effectiveness, a relatively common confounding factor in multiple genomic applications.

While DNA methylation patterns in cfDNA can potentially provide a wealth of biologically relevant information, to date few cfDNA-based assays have been approved by the FDA. Song *et al.* has demonstrated that methylation of SEPT9 is a highly sensitive biomarker in colorectal cancer (3), eventually developed into an FDA approved clinical test; Epi proColon test (Epigenomics AG) for colorectal cancer screening, which uses real-time PCR on bisulfite-converted plasma cfDNA to determine methylation status of the SEPT9 gene. Additionally, Klein *et al.* (GRAIL) recently published results showing cfDNA methylation can be used to distinguish patients with cancer from healthy individuals and to build models to predict cancer type, albeit with low sensitivity in early-stage cancers (16).

In most cases, diagnostic tests based on genomic data start with a discovery study where biologically significant features are identified genome wide between case and control. A more refined and targeted test is then constructed to evaluate features in as many people at as low a cost as possible. This second step is often extremely costly and time consuming as it can require developing a method from scratch. Furthermore, diagnostic tests require a high degree of accuracy and reproducibility (33). Here, we demonstrated that hybrid probe capture is both highly reproducible intra and inter-capture and highly accurate when compared to a WGBS reference. Importantly, aside from a period of probe design performed bioinformatically, the procedure itself did not need to be optimized and worked extremely well ‘right out of the box’. When combined with the scalability of this platform, hybrid probe capture could serve to enhance the design and feasibility of future DNA methylation diagnostic tests. In summary, the results of this study show that the myBaits Methyl-Seq system offers a cost-effective, reliable platform to profile DNA methylation, at a set of discrete custom regions, with potential applicability to liquid biopsies for cancer monitoring and recurrence risk.

## DATA AVAILABILITY

Beta value and coverage matrices (per base and per region) have been made available on Zenodo (https://doi.org/10.5281/zenodo.7374290).

## Supplementary Material

lqac099_Supplemental_Files

## Notes

Present address: Bodour Salhia, Department of Translational Genomics, University of Southern California, Los Angeles, CA 90033, USA.
