# Supplementary material for: Targeted DNA methylation from cell-free DNA using hybridization probe capture
Source: NAR Genom Bioinform. 2022 Dec 31;4(4):lqac099. doi: 10.1093/nargab/lqac099 (PMC9803870; doi:10.1093/nargab/lqac099)
Supplement: lqac099_Supplemental_Files [file lqac099_supplemental_files.zip › merge_SF.pdf]

Targeted DNA methylation from cell free DNA using hybrid probe capture

Supplementary Figures

July 10, 2022

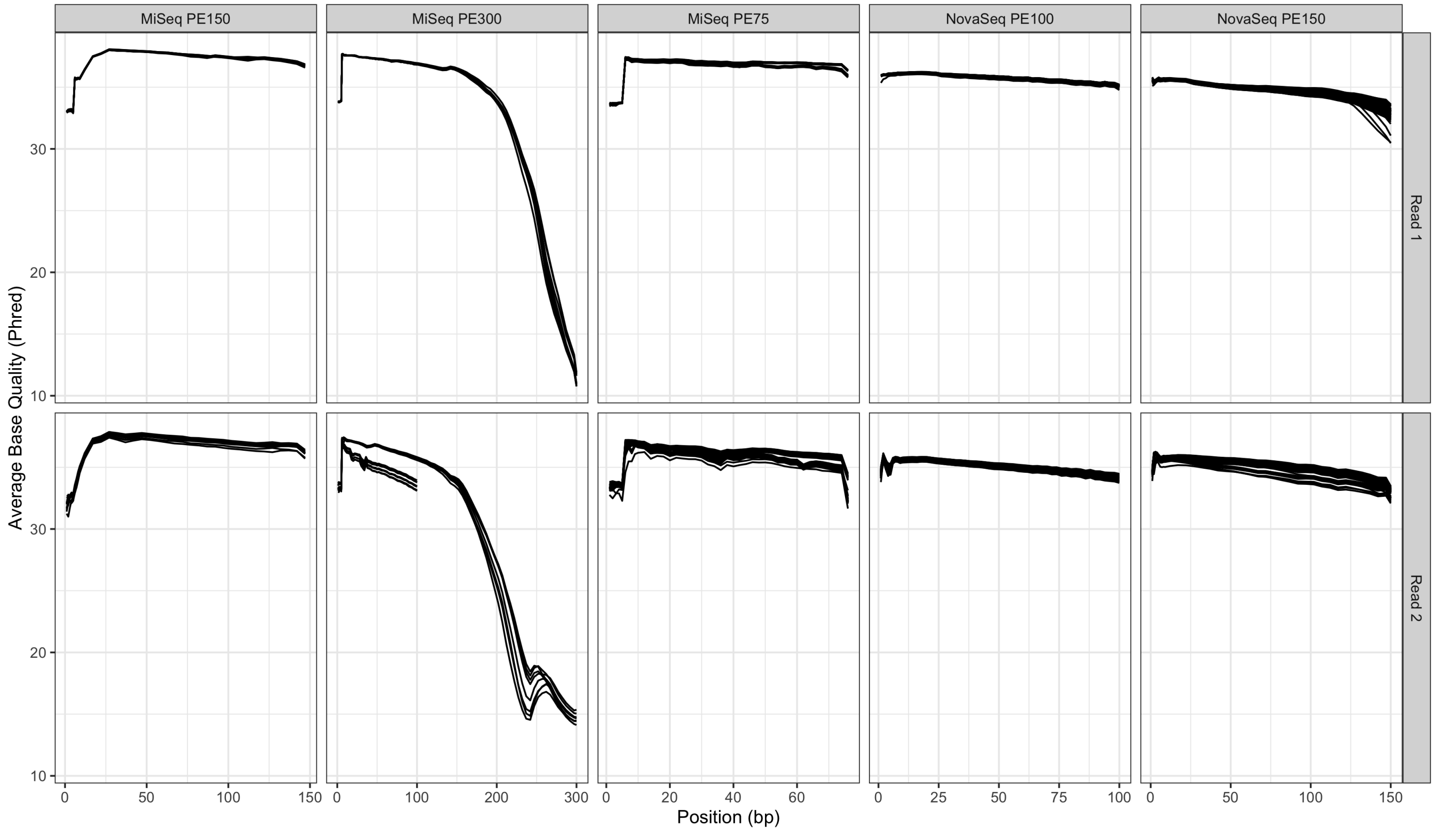

Supplemental Figure 1: FastQC per base sequence quality by read and sequencer/chemistry.

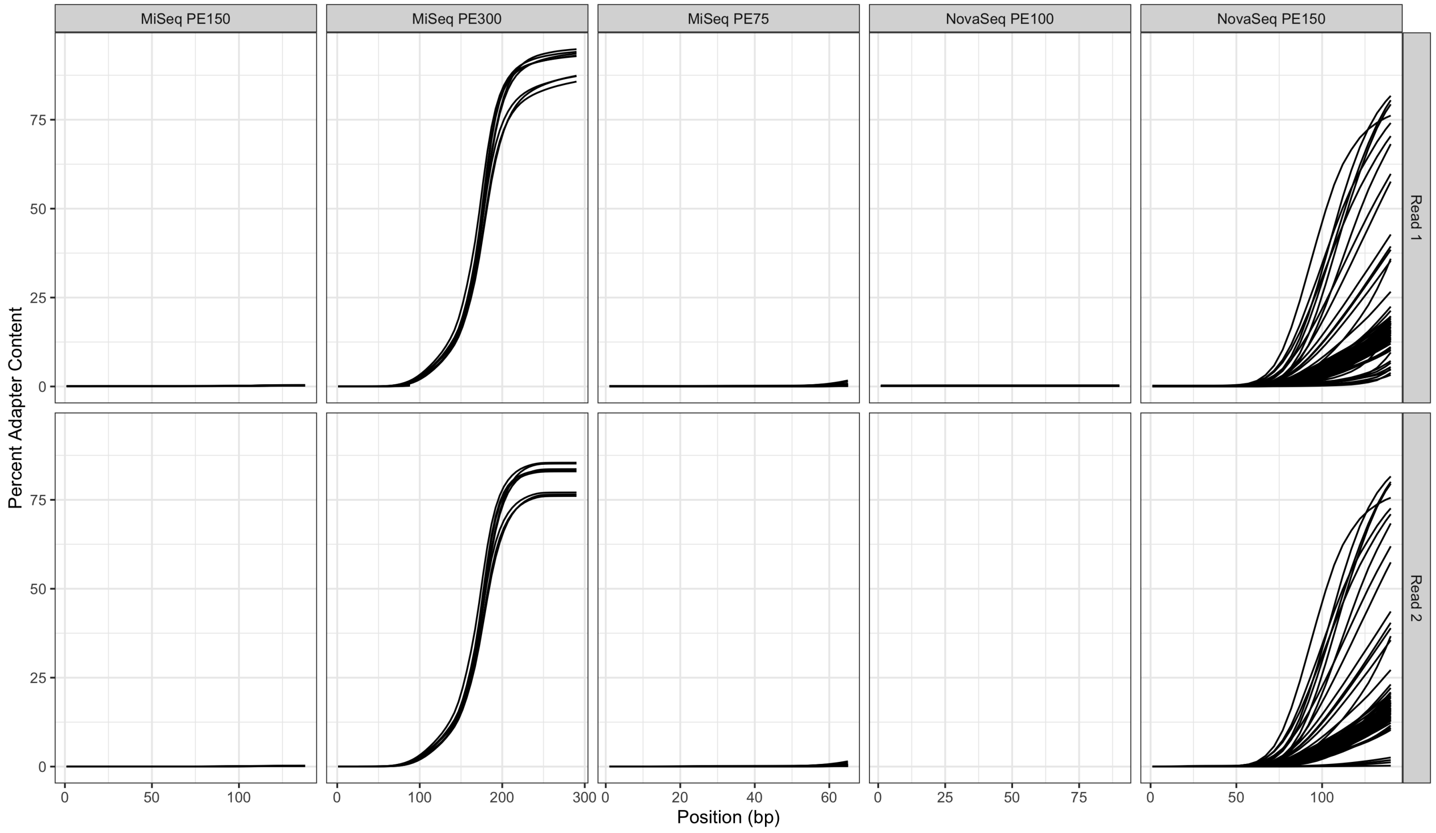

Supplemental Figure 2: FastQC per base adapter content by read and sequencer/chemistry.

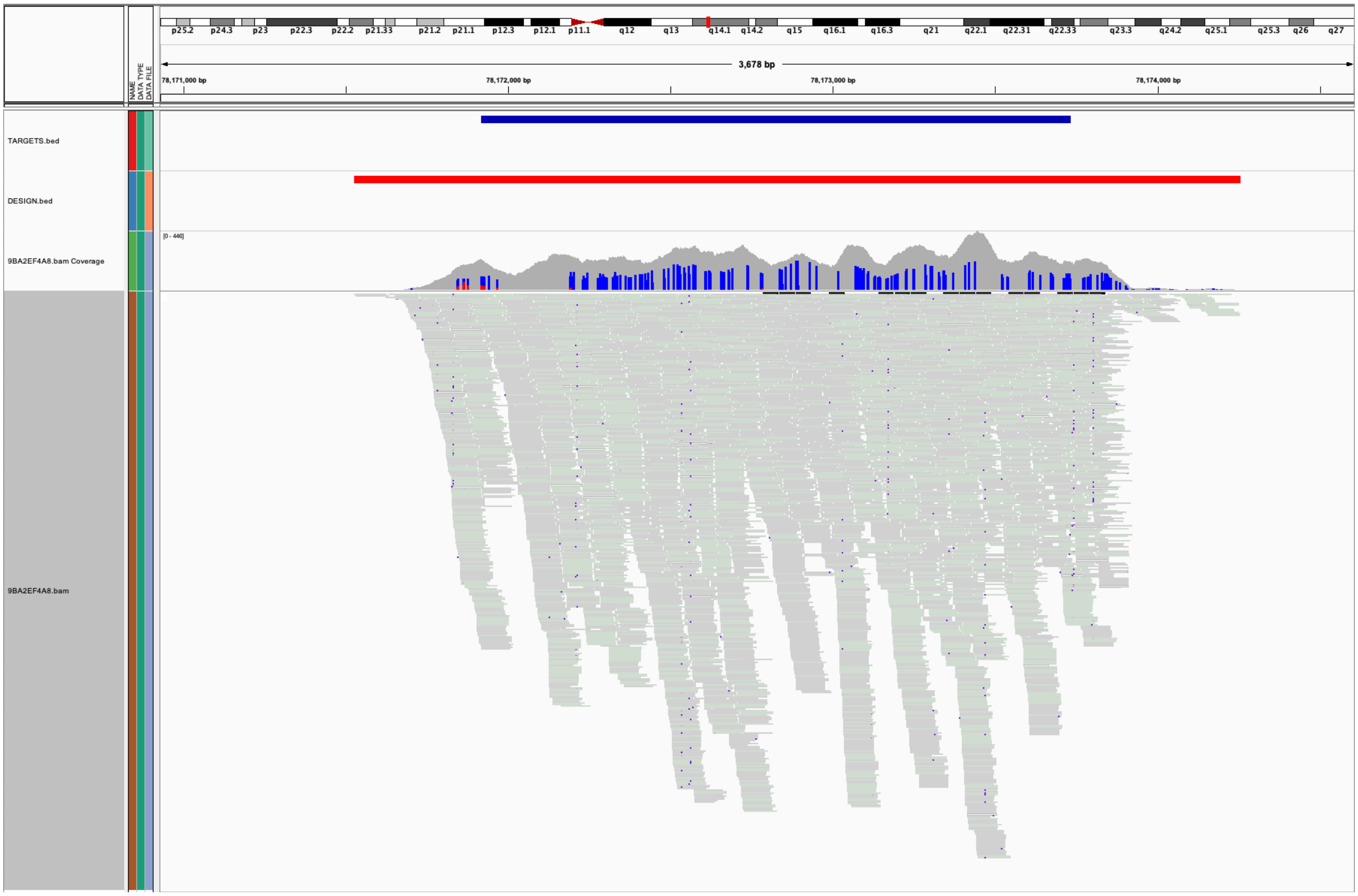

Supplemental Figure 3: Example of a typical hybrid probe capture target region.
In blue is the target region (the original DMR call). In red is the design region of the capture, with a small buffer upstream and downstream of the target region to ensure adequate coverage at the start and end. Reads from an example bam were included along with a coverage track (both grey).

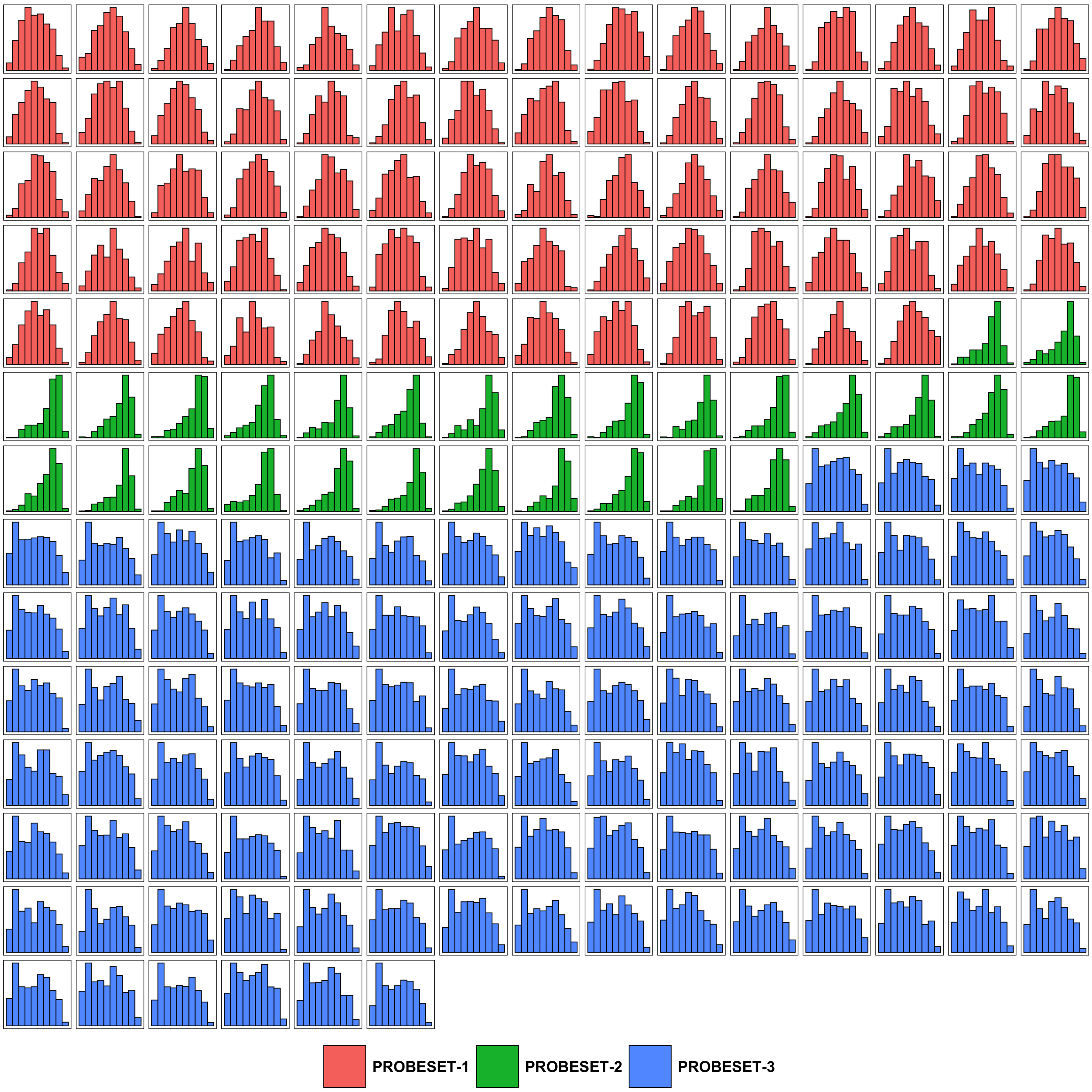

Supplemental Figure 4: Histogram of beta value distribution skewed distribution in samples run using probeset-2 Each histogram represents one sample, beta values from 0 to 1 span the x-axis in 10 bins.

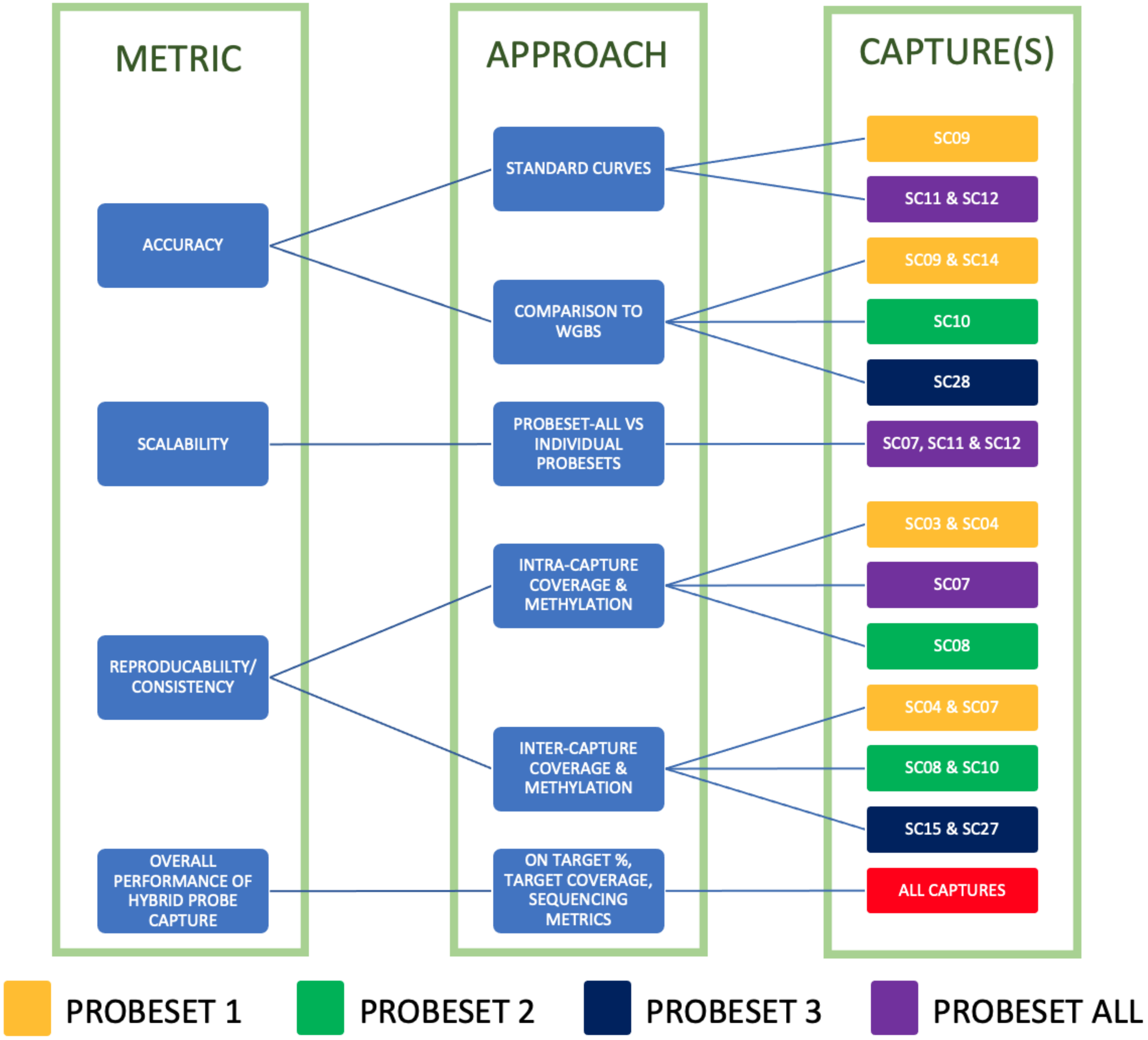

Supplemental Figure 5: Diagram of overall experimental design.
