# Supplementary material for: Targeted DNA methylation from cell-free DNA using hybridization probe capture
Source: NAR Genom Bioinform. 2022 Dec 31;4(4):lqac099. doi: 10.1093/nargab/lqac099 (PMC9803870; doi:10.1093/nargab/lqac099)
Supplement: lqac099_Supplemental_Files [file lqac099_supplemental_files.zip › supplementary_methods.docx]

**SUPPLEMENTARY METHODS**

**Whole genome bisulfite sequencing (WGBS)**

CfDNA and gDNA was used for whole genome bisulfite sequencing analysis (WGBS) as previously described (18). WGBS was performed on the following samples (Table S1): 1) plasma cfDNA from breast cancer (n = 5 pools consisting of a total 56 individuals across pools); 2) plasma cfDNA from healthy female individuals (n = 3 pools consisting of 39 individuals across pools); 3) plasma cfDNA from bladder cancer patients who relapsed after surgery (n = 5 pools consisting of 40 individuals); 4) plasma cfDNA from bladder cancer patients who did not relapse after surgery (n = 5 pools consisting of 46 individuals) and 5) samples from refractory paediatric cancers (n = 37 gDNA from tissue samples, 7 matching cfDNA samples) and their adjacent non-neoplastic tissue (n = 13). Directional bisulfite-converted libraries for paired-end sequencing were prepared using the Ovation Ultralow Methyl-Seq Library System (NuGen), using the manufacturer’s suggested protocol. Bisulfite conversion was performed using the EpiTect Fast DNA Bisulfite Kit (Qiagen). Post-library QC was performed on the 4200 Tapestation using D1000 High Sensitivity ScreenTapes (Agilent). Paired-end sequencing was performed on the Illumina NovaSeq 6000 platform using the S2 or S4 flowcell for a total read length of 2x150 bp.

**Feature selection from WGBS data for probe design**

To evaluate hybrid probe capture as a platform for targeted methylation analysis we designed probesets to differentially methylated regions (DMRs) from different sets of samples derived from patients with cancer and healthy individuals (Table S1). For this we used samples analysed by WGBS in the following comparisons: 1) 5 breast cancer plasma pools were compared against 3 female healthy pooled samples; 2) 5 pooled bladder cancer samples from patients with recurrence were compared to 5 bladder cancer pools from patients without recurrence; and 3) 37 gDNA samples (non-pooled) from recurrent or refractory paediatric cancer samples were compared to 13 adjacent normal tissues. For each of the breast, bladder and paediatric cancer datasets we identified DMRs using metilene as previously described (1). Metilene calls with false discovery rate corrected p-values < 0.05, and |Δβ| > 0.15 were considered DMRs and used for target assay design. The selected DMRs were used for probe design and each set referred to as probesets 1 (bladder cancer DMRs), 2 (paediatric cancer DMRs) and 3 (breast cancer DMRs). Probeset-1 was designed to 300 DMRs spanning 43,461bp containing 1731 CpGs, probeset-2 was designed to 402 DMRs spanning 163,426bp containing 3593 CpGs, and probeset-3 was designed to 713 DMRs spanning 282,950bp containing 11,570 CpGs.

**WGBS DMR annotation**

We annotated the genomic context of DMRs used for probe design using publicly available data from the UCSC genome browser. To define CpG islands, shores, and shelves, we downloaded the hg19 CpG island track from UCSC; CpG shores were defined as regions 0-2000bp upstream and downstream of a CpG island; CpG shelves were defined as regions 2000-4000bp upstream and downstream of a CpG island, as previously described (2). In cases where a DMR overlapped multiple features it was tabulated according to the following hierarchy: CpG Island > CpG shore > CpG shelf. If a DMR did not overlap any feature it was tabulated as ‘Open Sea’.

To define functional regions, we used the hg19 knownGene table from UCSC to define exons, introns, and promoters. We used the hg19 GeneHancer track from UCSC to define enhancer regions (3). In cases where a DMR overlapped multiple features it was tabulated according to the following hierarchy: promoter > exon > enhancer > intron. If a DMR did not overlap any feature it was tabulated as ‘Intergenic’.

**REFERENCES**

1. Legendre, C., Gooden, G.C., Johnson, K., Martinez, R.A., Liang, W.S. and Salhia, B. (2015) Whole-genome bisulfite sequencing of cell-free DNA identifies signature associated with metastatic breast cancer. *Clin Epigenetics*, **7**, 100.

2. Legendre, C.R., Demeure, M.J., Whitsett, T.G., Gooden, G.C., Bussey, K.J., Jung, S., Waibhav, T., Kim, S. and Salhia, B. (2016) Pathway Implications of Aberrant Global Methylation in Adrenocortical Cancer. *PLoS One*, **11**, e0150629.

3. Fishilevich, S., Nudel, R., Rappaport, N., Hadar, R., Plaschkes, I., Iny Stein, T., Rosen, N., Kohn, A., Twik, M., Safran, M. *et al.* (2017) GeneHancer: genome-wide integration of enhancers and target genes in GeneCards. *Database (Oxford)*, **2017**.
